# Impact of a toothpaste with microcrystalline hydroxyapatite on the occurrence of early childhood caries: a 1-year randomized clinical trial

**DOI:** 10.1038/s41598-021-81112-y

**Published:** 2021-01-29

**Authors:** Elzbieta Paszynska, Malgorzata Pawinska, Maria Gawriolek, Inga Kaminska, Justyna Otulakowska-Skrzynska, Grazyna Marczuk-Kolada, Szymon Rzatowski, Katarzyna Sokolowska, Aneta Olszewska, Ulrich Schlagenhauf, Theodor W. May, Bennett T. Amaechi, Elzbieta Luczaj-Cepowicz

**Affiliations:** 1 Department of Integrated Dentistry, Poznan University of Medical Sciences, Poznan, Poland; 2 Department of Integrated Dentistry, Medical University of Bialystok, Białystok, Poland; 3 Department of Pedodontics, Medical University of Bialystok, Białystok, Poland; 4 Department of Facial Malformation, Pediatric Dentistry Clinic, Poznan University of Medical Sciences, Poznan, Poland; 5 Department of Conservative Dentistry and Periodontology, University Hospital Wuerzburg, Wuerzburg, Germany; 6 Society for Biometrics and Psychometrics, Bielefeld, Germany; 7 Department of Comprehensive Dentistry, School of Dentistry, University of Texas Health San Antonio, San Antonio, TX USA

**Keywords:** Diseases, Medical research

## Abstract

The aim of this trial was to determine whether a toothpaste with microcrystalline hydroxyapatite is not inferior to a fluoride toothpaste in prevention of caries in children. This double-blinded randomized control trial compared two toothpastes regarding the occurrence of caries lesions using *International Caries Detection and Assessment System* (ICDAS) ≥ code 1 on the primary dentition within 336 days. The test group used a fluoride-free hydroxyapatite toothpaste three times daily while control group used a toothpaste with fluoride. 207 children were included in the intention-to-treat analysis; 177 of them finished the study per protocol. An increase in caries ICDAS ≥ code 1 per tooth was observed in 72.7% of the hydroxyapatite-group (*n* = 88), compared with 74.2% of the fluoride-group (*n* = 89). The exact one-sided upper 95% confidence limit for the difference in proportion of participants with ICDAS increase ≥ 1 (-1.4%) was 9.8%, which is below the non-inferiority margin of 20% demonstrating non-inferiority of hydroxyapatite compared to the fluoride control toothpaste. This RCT showed for the first time, that in children, the impact of the daily use of a toothpaste with microcrystalline hydroxyapatite on enamel caries progression in the primary dentition is not inferior to a fluoride control toothpaste (Clinical Trials NCT03553966).

## Introduction

Early childhood caries (ECC) is still one of the most prevalent diseases worldwide^1–3^. Children of any socioeconomic status can be affected by ECC^4,5^. Although a general trend in caries decline has been observed^6,7^, current data show that caries is still a highly prevalent disease^8–11^. In Poland, for example, 76.9% of 5-year-old children and 89.4% of 7-year-old children are still affected by caries^8^. Even in Germany with a well-established health care system and a long-term record of declining caries prevalence figures still 13.7% of 3-year-old, 43.6% of 6–7-year-old, and also 21.2% of 12-year-old children have at least one tooth with a caries experience^9^. Other developed countries like Australia and the USA show a comparable high prevalence of ECC^10,11^.

The prevention of dental caries in children and adults follows a multifactorial approach^12^. Besides promoting a healthy, low-sugar diet^13^, a thorough preventive oral health care, i.e. toothbrushing with toothpaste, at home is advised to reduce the caries-risk^14^.

Toothpastes should promote remineralization and inhibit demineralization of enamel and dentin to prevent dental caries^14,15^. Thus, fluoride provided as amine fluoride (e.g. Olaflur; C_27_H_60_F_2_N_2_O_3_), sodium fluoride (NaF), sodium monofluorophosphate (Na_2_PO_3_F), or stannous fluoride (SnF_2_) are well-known for their caries preventing effect and consequently they are frequently used in many toothpaste formulations^6,14,16–18^. However, it is known that infants and toddlers swallow a substantial proportion of the applied toothpaste during toothbrushing^19–24^, which can increase the systemic uptake of fluorides beyond a threshold associated with the occurrence of dental fluorosis and other unwanted side effects^19,25^. Therefore, in many countries toothpastes for children contain a reduced amount of fluorides compared to toothpastes for adults^17^.

Besides fluorides, another approach for caries prevention is to focus on biomimetic and bio-inspired agents promoting remineralization and inhibiting demineralization of the dental hard tissue. One of these biomimetic agents is particulate hydroxyapatite (HAP; a calcium phosphate mineral; Ca_5_(PO_4_)_3_(OH))^26–32^. HAP has been studied in different fields of preventive oral health care^14,29–47^. Unlike fluoride, the accidental swallowing of HAP as a toothpaste ingredient is not associated with any relevant systemic health risks such as fluorosis, as hydroxyapatite is the main inorganic component of all human hard tissues, like teeth and bones^48^.

In Germany, Italy, Japan, and other countries worldwide, HAP toothpastes are commercially available for many years^14,36,49^. The anti-caries efficacy of HAP-toothpastes could be shown e.g. in a placebo-controlled clinical trial in Japanese school children^38^. Based on this study, HAP was approved as anti-caries agent in Japan in 1993^14^.

The caries-preventing efficacy of a HAP-toothpaste compared to fluorides has been evaluated in a randomized controlled 6-month trial^31^ using *The International Caries Detection and Assessment System* (ICDAS)^31,50,51^. This study showed that in orthodontic patients the daily use of a HAP-toothpaste was not inferior to a fluoride toothpaste with 1400 ppm fluoride (amine fluoride and stannous fluoride) with respect to caries progression^31^. Due to the antibacterial fluoride counter ion (i.e. the ammonium salt), amine fluoride shows, in contrast to sodium fluoride and sodium monofluorophosphate, additional antibacterial/antibiofilm properties^52–54^. Additionally, a recent in situ study showed an effective remineralization of early caries lesions by a HAP toothpaste. Here, the remineralization effect was comparable to a toothpaste with 500 ppm fluoride (provided as amine fluoride). While the fluoride toothpaste showed a remineralization of mainly the surface-layer, the HAP toothpaste remineralized also the deeper enamel layers as revealed by transverse microradiography images^32^. While the optimal fluoride concentration of toothpastes for children is still subject to discussions (i.e. caries preventing efficacy vs. fluorosis-risk), the caries-inhibiting efficacy of children’s toothpastes containing 500 ppm fluoride has been demonstrated by several clinical studies^16,17,55,56^.

The objective of this clinical trial was to compare, for the very first time, the impact of the daily use of a HAP-containing children’s toothpaste on the development of enamel caries in the primary dentition with use of a fluoride control toothpaste with proven caries preventive efficacy. Enamel caries development was monitored according to the criteria of the *International Caries Detection and Assessment System* (ICDAS II)^50,51^. The hypothesis of this study was that the impact of the evaluated fluoride-free biomimetic HAP-containing children's toothpaste on enamel caries development in the primary dentition is not inferior to the impact of a fluoride control toothpaste.

## Subjects and methods

### Study design and test centers

This multicenter, double-blind, randomized, active-controlled, parallel-group 336 days study was performed in children with an initial age of 3–7 years at the University Hospitals of Poznan and Bialystok, in Poland. The trial was approved by the ethics committees of the University Hospitals of Poznan and Bialystok and was registered at ClinicalTrials.gov (NCT03553966). Planning and conduct of the study were in accordance to the declaration of Helsinki and the principles of *Good clinical practice* (GCP). During the study, an external study monitor (Dr. Egmont Zieseniss, Inpharm-Consulting, Dortmund, Germany) regularly reviewed the case report forms to verify completeness, plausibility, data consistency, protocol adherence, and the progress of enrolment. He also ensured that study supplies were being stored, dispensed, and accounted for according to specifications.

### Primary and secondary endpoints

#### Primary endpoint

Primary endpoint was the proportion of study subjects experiencing the development of at least one new enamel caries lesion ≥ ICDAS code 1 or the progression of an existing enamel caries lesion by at least one ICDAS score on any of the evaluated primary molars during the observation period of 336 days. The inspected areas were all enamel surfaces of the primary molars (i.e. buccal, distal, lingual, mesial, occlusal). In this study, ICDAS II was applied based on criteria described by Ismail et al.^50^.

An enamel caries lesion with ICDAS code ≥ 1 or the progression of an existing enamel caries lesion by at least one ICDAS code in a given study participant was documented, when at least one of the assessed primary molars fulfilled one of the following conditions:

1. ICDAS code 0 on all surfaces at baseline [visit 2] AND ICDAS code ≥ 1 on at least one surface at follow-up [visit 3 to visit 6].
2. ICDAS code 1 (on at least one surface) at baseline [visit 2] AND ICDAS code ≥ 2 on at least one (not necessarily the same) surface at follow-up [visit 3 through visit 6].
3. ICDAS code 2 (on at least one surface) at baseline [visit 2] AND ICDAS code ≥ 3 on at least one (not necessarily the same) surface at follow-up [visit 3 to visit 6].
4. No filling (restoration) at baseline [visit 2] AND presence of a restoration (i.e. treatment of a caries lesion of ICDAS code ≥ 3) at the follow-ups [visit 3 to visit 6]

#### Secondary endpoints

Secondary endpoints were:

1. The percentage of study subjects experiencing the development of at least one new enamel caries lesions ≥ ICDAS code 2 on any of the evaluated primary molars (i.e. buccal, distal, lingual, mesial, occlusal surfaces) during the observation period (336 days),
2. The changes in the coverage of the assessed primary molars with bacterial plaque according to the criteria of the Plaque Control Record^57^, and
3. The changes in the status of gingival health of the assessed primary molars according to the criteria of the Modified Gingival Index^58^.

### Inclusion criteria and exclusion criteria

The following exclusion and inclusion criteria were applied:

#### Inclusion criteria

- Age 3–7 years
- Complete set of fully erupted primary molars (teeth 55, 54, 64, 65, 75, 74, 84, 85)
- Presence of a restoration (filling) due to caries on at least 1 primary molar
- Minimum of 4 primary molars without a restoration or sealed fissure/pit

#### Exclusion criteria

- Untreated caries lesions of ICDAS code 3–6
- Known hypersensitivity to one of the ingredients of the toothpastes to be tested
- Systemic disorders interfering with salivary function or flow
- Regular medication intake interfering with salivary function or flow
- Need for antibiotic prophylaxis during dental treatments
- Participation in any other clinical study within the past 3 months or ongoing
- Lack of intellectual or physical ability to follow the instructions of the study protocol
- Any other reason that, in the opinion of the investigator, excludes the subject from eligibility for study participation

### Treatment

Both test toothpaste (HAP) and control toothpaste (fluoride) were handed out to the parents of the study participants by study nurses, not being involved in the clinical assessment of the study parameters, using a computer-generated randomization list. Randomization was performed separately for each study center and was stratified for the number of restored primary molars at baseline.

- *Stratum A:* Baseline number of primary molars with restorations ≥ 1 ≤   2.
- *Stratum B:* Baseline number of primary molars with restorations ≥ 3 ≤   4.

### Toothpastes and toothbrushes

To ensure blinding, both study toothpastes (HAP test, fluoride control) were provided in neutral plastic tubes of identical shape and color but differentiated with code numbers known to only the manufacturer. Both toothpastes were manufactured by a *Good Manufacturing Practice* (GMP) certified external laboratory.

The test toothpaste with 10% microcrystalline HAP^34^ was identical in composition to a commercially available product (Kinder Karex Zahnpasta, Dr. Kurt Wolff GmbH & Co. KG, Bielefeld, Germany) and contained the following ingredients:

Aqua, Hydrogenated Starch Hydrolysate, Hydrated Silica, Hydroxyapatite, Xylitol, Silica, Cellulose Gum, Aroma, 1,2-Hexanediol, Caprylyl Glycol, Sodium Methyl Cocoyl Taurate, Sodium Cocoyl Glycinate, Sodium Sulfate, Limonene (pH in 1:10 aqueous solution was 7.7).

The fluoride control toothpaste with amine fluoride (500 ppm F^-^) was also a commercially available product (elmex Kinder-Zahnpasta CP GABA GmbH, Hamburg, Germany) and contained the following ingredients in addition to amine fluoride: Aqua, Sorbitol, Hydrated Silica, Hydroxyethylcellulose, CI 77,891, Cocamidopropyl Betaine, Olaflur, Aroma, Saccharin, Limonene (pH in 1:10 aqueous solution was 4.8).

#### Toothbrushes

Next to the assigned toothpastes the study participants were also provided with a standardized electric toothbrush (Braun Oral-B Stage Power, P&G, Schwalbach, Germany).

Instructions were given to parents of the participants to brush the teeth of their children with the assigned toothpaste and the provided toothbrush for 3 min in the morning and in the evening over the observation period of 336 days.

Additionally, all study participants brushed their teeth themselves at noon for 3 min with the assigned experimental toothpaste using a manual children's toothbrush (elmex Kinder-Zahnbürste, CP GABA GmbH, Hamburg, Germany) and applying a horizontal scrub technique under the supervision of an adult. In total, brushing with the study toothpastes was performed 3 × daily. A brushing diary was used to monitor toothbrushing frequency.

Note that besides the study toothpastes, no other fluoride- and/or antiseptics-containing dental care products (mouthwashes, gels etc.) were used during this study. Furthermore, no professional tooth cleaning was performed.

### Course of the study

During the course of the study 6 visits were scheduled at the clinics in Poznan and Bialystok, Poland.

#### Visit 1 (screening): 0–63 days before study start

Individuals potentially eligible for study participation and their parents were informed by the investigators about the aims, significance, and risks of study participation by a written patient information form and face to face interviews. Before study inclusion, the willingness of the child and the parents to properly follow the study protocol for the next 336 days was assessed. A child was included as study participant only after the parents had given their written informed consent. After informed consent was obtained, an initial examination took place to screen the potential subject for study eligibility (inclusion and exclusion criteria) and to document the subject’s demographic data.

#### Visit 2 (baseline): study day 0, enrollment in the study, collection of baseline data

In case Visit 1 dated back more than 7 days, investigators reconfirmed that inclusion and exclusion criteria did not change and patients were still eligible for inclusion in the study.

Assessment of the 3 study parameters was done on all surfaces of the 8 molars in this following sequence:

1. Modified Gingival Index (GI): Gingivitis was assessed visually without touching the gingiva on the buccal and lingual marginal gingivae and interdental papillae of the included 8 primary molars. The gingiva was segmented into 6 sites per tooth (mesio-buccal, buccal, disto-buccal and mesio-lingual, lingual, disto-lingual), and gingival inflammation was recorded at each tooth site on a scale of 0 to 4 as described Lobene et al.^58^:
  0—Normal (absence of inflammation)
  1—Mild inflammation (slight change in color, little change in texture) of any portion of the gingival unit
  2—Mild inflammation of the entire gingival unit
  3—Moderate inflammation (moderate glazing, redness, edema, and/or hypertrophy) of the gingival unit
  4—Severe inflammation (marked redness and edema/hypertrophy, spontaneous bleeding, or ulceration) of the gingival unit
2. Plaque Control Record (PCR): Then PCR scores were assessed by touching the tooth surfaces with a blunt periodontal probe to evaluate the coverage of the assessed primary molars with bacterial plaque^57^.
3. Afterwards, thorough teeth cleaning was performed by an experienced dentist in order to remove all dental plaque from the tooth surfaces. Professional teeth cleaning was not performed, as primary teeth and gingiva of those young children are very sensitive. Tooth brushing with a dentist was performed to ensure that all children started at the same baseline with respect to the coverage of the assessed primary molars with bacterial plaque.
4. Caries status (ICDAS): Caries assessment by ICDAS required the removal of adherent plaque by a toothbrush (see above).
  The teeth were first examined wet, then the surfaces were dried for 5 s with a dental air–water syringe, and again examined dry. All levels of caries lesions ranging from initial (non-cavitated) to cavitated lesions were visually identify on all surfaces (i.e. buccal, distal, lingual, mesial, occlusal surfaces) of each included primary molars, using the ICDAS II criteria (Scores 0–6) as described by Ismail et al.^50^:
  Score 0: sound tooth surface;
  Score 1: first visual change (opacity or discoloration) in enamel hardly visible on the wet surface but distinctly visible after air drying;
  Score 2: distinct visual change (opacity or discoloration) in enamel, visible without air drying;
  Score 3: localized enamel breakdown without visible dentin;
  Score 4: underlying dark shadow from dentin without cavitation;
  Score 5: distinct cavity with visible dentin;
  Score 6: extensive distinct cavity with visible dentin.
5. After assessment of GI, PCR, and ICDAS score by the examiner, the study subjects received an electric toothbrush (for brushing in the morning and in the evening), a manual toothbrush (for brushing at noon), and the allocated toothpaste (test or control) from a trained study nurse not involved in the preceding recording of GI, PCR and ICDAS. Proper use of the assigned electric toothbrush, the manual toothbrush, and the issued toothpaste were also instructed by this study nurse or a dentist not involved in the clinical examinations. To confirm the subjects’ understanding of the brushing instructions, they were requested to perform their first brushing episode there in the clinic under the supervision of the study nurse. Finally, subjects/parents received a brushing diary and new appointment date for the first follow-up visit (v*isit 3*) at 84 days (± 14 days).

#### Visit 3: study day 84 (± 14 days), 1st follow-up examination; visit 4: Study day 168 (± 14 days), 2nd follow-up examination, and visit 5: study day 252 (± 14 days), 3rd follow-up examination.

At each follow-up visit, the GI, PCR and ICDAS were reassessed as described for the baseline visit. Subsequently, a study nurse not involved in the assessment of these study parameters (GI, PCR, ICDAS) collected back the used toothpaste tubes and toothbrushes given at the baseline visit, and supplied the subjects with new manual toothbrush, a new brushing head for the electric toothbrush, and a new supply of the assigned experimental toothpaste (test or control) for the next 84 days. Furthermore, for all visits, the study nurse checked the efficacy of the oral hygiene efforts (e.g. plaque removal) of the subjects by supervising another brushing episode there in the clinic, and if necessary, re-trained the subjects on effective brushing technique. Finally, subjects/parents received a new brushing diary and a new appointment for the subsequent visit.

#### *Visit 6: study day 336 (*± *14 days), final visit*

336 days after baseline visit, the GI, PCR, and ICDAS assessments were repeated as described for the baseline visit. Subsequently a study nurse not involved in the assessment of the study parameters (GI, PCR, ICDAS) collected back the used toothpaste tubes and toothbrushes given at the baseline visit. Subjects’ parents were informed that subjects may now resume using their usual toothpaste.

### Sample size calculation

It was assumed that the primary endpoint (development of at least one new enamel caries lesion ≥ ICDAS code 1 or progression of an existing enamel caries lesion by at least ICDAS code ≥ 1) would occur in about 70% of the study subjects during the observation period (48 weeks)^59^. The non-inferiority margin Δ was set to 20%^31^. A sample size of 2 × 75 study subjects was calculated to be sufficient to reject the null hypothesis that the test toothpaste is inferior to the control toothpaste, using a non-inferiority margin of Δ = 20% for the primary endpoint and one-sided, exact Fisher’s Test (α = 5%, power = 90%). Assuming a drop-out rate of 25% of the study population, a total of 200 study subjects had to be included in the study to reach at least 150 study subjects for the analysis.

### Statistical analysis

The primary outcome measure was analyzed primarily for the per protocol (PP) population and repeated for sensitivity reasons for the intent-to-treat (ITT) population. For the primary endpoint, non-inferiority was claimed if the exact one-sided upper 95% confidence limit (CI) for the corresponding difference between test and control toothpaste was less than the pre-set inferiority margin Δ (difference) of ≤ 20% [≤ 0.20]^60^. In addition, logistic regression analyses were performed with the primary endpoint and the secondary endpoint of development of a new enamel caries lesion with ICDAS code ≥ 2 as dependent variable and toothpaste, center, stratum (1–2 filled molars vs. 3–4 filled molars), and age as independent variables (covariates). Analyses of covariance (ANCOVA) and non-parametric tests (two-sided Mann–Whitney test for between-group comparisons and Friedman tests for within-group comparisons) were performed for the secondary endpoints PCR and GI. IBM SPSS Statistics, version 25.0 (IBM Corp., Armonk, N.Y., USA) and SAS 9.4 software package (SAS Institute, Cary, NC, USA) were used for the statistical evaluations. Figures 2, 3, 4 and 5 were produced using the open software R (ggplot2)^61^.

Note that the planned duration of the study was 336 day (48 weeks). But, in case that ≥ 70% of study participants would have experienced the primary study outcome (i.e. development of an enamel caries lesion ≥ ICDAS code 1 on one or more of the evaluated primary molars) already at Visit 4 (study day 168 [± 7 days], 2^nd^ follow-up examination), the study would have been finished prematurely. The blinded interim analysis revealed that < 70% of study participants experienced the primary study outcome. Thus, the study was not prematurely finished.

### Blinding and randomization

The study was designed as a double-blind trial. Using block randomization (provided by T.W.M.) with a block size of 4, a random list was generated to code the test and control toothpaste tubes with consecutive unique identification numbers. The randomization of toothpaste assignment was stratified by the study center and stratum (number of filled (restored) molars). Distribution of the experimental toothpastes to the study patients followed the sequence of the identification numbers and was performed by trained study nurses not involved in the examination of the study participants.

#### Inter-examiner reliability (ICDAS)

All examiners (Poznan: 4 dentists, Bialystok: 5 dentists) involved in the practical assessment of ICDAS were trained before the onset of the trial by using the ICDAS e-learning course at the ICDAS website (www.iccms-web.com) as well as by intensive practical training of ICDAS assessment under the guidance of an experienced cariologist. Scoring skills of the investigators were trained at the start of study and retrained twice more during the course of the study using an additional internet-based ICDAS training tool (specially developed for this study; assessment of 26 clinical photographs of teeth with different ICDAS-scores; random sequence). Based on the results of this online tool, the linear weighted kappa coefficient was calculated as measure of the inter-rater reliability of ordinal scaled categories (ICDAS codes) between two raters. The inter-examiner reliability was calculated using the open source statistical software R with the package IRR (kappa2; option: weight = “equal “). In addition, the squared weighted kappa coefficients were calculated (kappa2; option: weight = "squared").

## Results

### Subjects

The demographic data of the per protocol (PP) population are shown in Table 1.

**Table 1:** Demographic data of the per protocol (PP) population.

	Treatment					
	HAP toothpaste			Fluoride toothpaste		
	Stratum			Stratum		
	A	B	Total	A	B	Total
**Sex**						
*Female*						
N	36	12	48	40	12	52
%	52.9%	60.0%	54.5%	63.5%	48.0%	59.1%
*Male*						
N	32	8	40	23	13	36
%	47.1%	40.0%	45.5%	36.5%	52.0%	40.9%
**Age (months)**						
*Mean*						
	58.2	62.5	59.2	60.8	66.2	62.3

A total of 214 children were included in the study: 107 (50%) of them were assigned to the HAP toothpaste group and 107 (50%) to the fluoride toothpaste group. 202 (94.4%) subjects completed visit V6: HAP: *n* = 101 (50%), fluoride: *n* = 101 (50%). The study was prematurely terminated in 12 subjects (HAP: *n* = 6, fluoride: *n* = 6) for various reasons (Table 8). Due to major violations of the window for protocol-defined visits (> ± 4 weeks), 17 subjects (HAP: *n* = 9, fluoride: *n* = 8) were excluded from the per-protocol analysis. Furthermore, 8 subjects (HAP: *n* = 4, fluoride: *n* = 4) were excluded due to non-compliance from the per-protocol analysis. Non-compliance was defined based on subject diaries if application of toothpaste was ≤ 1 × daily in > 10% of study days (except last month of the study). Thus, 37 (17.3%) subjects were excluded from PP-population: HAP: *n* = 19, fluoride: *n* = 18. In total, 177 (82.7%) subjects were included in the PP set: HAP: n = 88 (82.2%), fluoride: n = 89 (83.2%).

All subjects who applied the test or control toothpaste at least once were included in the intention-to-treat analysis set (ITT). Missing values were replaced by last-observation-carried-forward (LOCF) provided that any follow-up visits (V3-V6) were performed. In 7 subjects the study was terminated already at visit V2 (Baseline), i.e. these subjects were not included in the ITT-analyses (HAP: *n* = 4, fluoride: *n* = 3). In total, 207 subjects were included in the ITT set: HAP: *n* = 103, fluoride: *n* = 104. The primary endpoint was analyzed for the PP set and, in addition, repeated for sensitivity reasons for the ITT set.

### Patient flow chart

**Figure 1:**
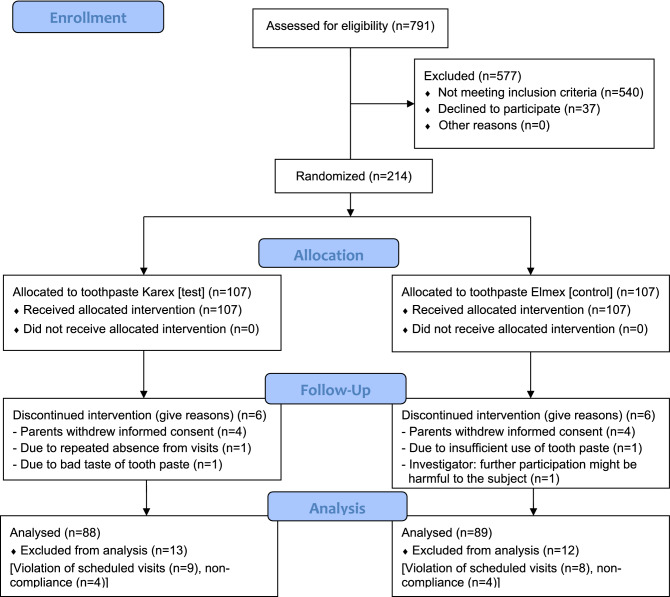
Patient flow chart.

The “Patient Flow Chart” according to the CONSORT Statement is shown in Fig. 1.

### Primary endpoint

Table 2 summarizes the percentage of subjects who developed enamel caries lesions with ICDAS code ≥ 1 or a progression of an existing enamel caries lesion by at least one ICDAS code.

**Table 2:** Development of enamel caries lesions with ICDAS code ≥ 1 or the progression of an existing enamel caries lesion by at least one ICDAS code in the PP-population and confidence intervals for the “risk difference”.

PP-population	Treatment/toothpaste					
	HAP (test)		Fluoride (control)		Total	
	n	%	n	%	n	%
**ICDAS increase ≥ 1 per tooth**						
No	24	27.3%	23	25.8%	47	26.6%
Yes	64	72.7%	66	74.2%	130	73.4%
Total	88	100.0%	89	100.0%	177	100.0%

Primary endpoints	Population	Difference HAP-fluoride	Lower CI95% one-sided	Upper CI95% one-sided	Lower CI95% two-sided	Upper CI95% two-sided
**Confidence Intervals for the “risk difference” HAP – fluoride**						
ICDAS increase ≥ 1 per tooth	PP	− 1.4%	− .12.4%	9.5%	− 14.4%	11.6%

CI95%, confidence interval; PP, per protocol; HAP, hydroxyapatite.

**Figure 2:**
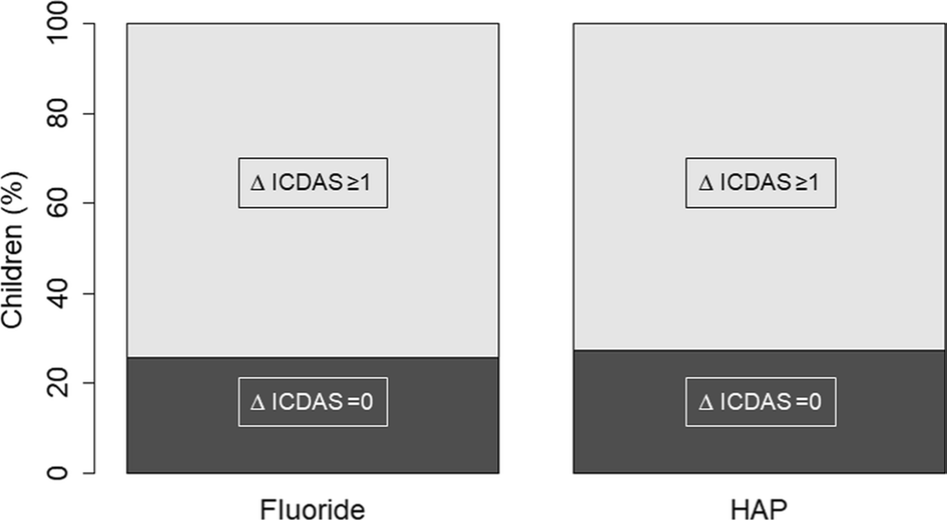
Increase in ICDAS ≥ ∆1 in the fluoride, and HAP-group. In both groups, the proportion of children who developed at least on one tooth a caries lesion of ICDAS ≥ ∆1 is not significantly different (more details are shown in Table 2).

The exact one-sided upper 95% CI limit for the difference in proportion of subjects with ICDAS increase ≥ 1, per tooth as well as per surface was 9.5% which is clearly below the non-inferiority margin 20% (PP analysis) (Fig. 2). Thus, the test toothpaste (HAP) can be considered non-inferior to the control toothpaste (fluoride). This is also true for the exact two-sided upper 95% limit (11.6%). Superiority cannot be assumed as the confidence intervals are not below zero. The results of the ITT-analysis/Table 3) confirmed the results of the PP-analysis (Table 2).

**Table 3:** Development of enamel caries lesions with ICDAS code ≥ 1 or the progression of an existing enamel caries lesion by at least one ICDAS code in the ITT-population and confidence intervals for the “risk difference”.

ITT-population	Treatment/toothpaste					
	HAP (test)		Fluoride (control)		Total	
	n	% (column)	n	% (column)	n	% (column)
**ICDAS increase ≥ 1 per tooth**						
No	26	25.2%	26	25.0%	52	25.1%
Yes	77	74.8%	78	75.0%	155	74.9%
Total	103	100.0%	104	100.0%	207	100.0%

Primary endpoints	Population	Difference HAP-fluoride	Lower CI95% one-sided	Upper CI95% one-sided	Lower CI95% two-sided	Upper CI95% two-sided
**Confidence intervals for the “risk difference” HAP-Fluoride**						
ICDAS increase ≥ 1 per tooth (Version 1)	ITT	− 0.2%	− 10.2%	9.7%	− 12.1%	11.6%

CI95%, confidence interval; ITT, intent-to-treat; HAP, hydroxyapatite.

In addition, a logistic regression analysis was performed with the primary endpoint as dependent variable and toothpaste, center, number of filled molars, and age as independent variables (covariates). The results for the PP population confirmed that the “risk” of development of new enamel caries lesion on ICDAS ≥ code 1 was not significantly dependent on “toothpaste” (HAP vs. fluoride).

### Secondary endpoints

#### Development of at least one new enamel caries lesions with ICDAS code ≥ 2

The proportion of subjects with development of at least one new enamel caries lesion with ICDAS ≥ code 2 up to visit 6 (56.5%) was lower than the proportion of subjects with development of enamel caries lesions with ICDAS ≥ code 1 or the progression of an existing enamel caries lesion by at least increase in ICDAS ≥ code 1 (74.9%) (Tables 3 and 4).

**Table 4:** Development of at least one enamel caries lesions with ICDAS code ≥ 2 in the PP-population.

PP-population	Treatment/toothpaste					
	HAP		Fluoride		Total	
	n	%	n	%	N	%
**Proportion of subjects with ICDAS increase ≥ 2 in at least one tooth up to visit 6 (after 48 weeks)**						
No	40	45.5%	37	41.6%	77	43.5%
yes	48	54.5%	52	58.4%	100	56.5%
Total	88	100.0%	89	100.0%	177	100.0%

Secondary endpoint	Population	Difference HAP-fluoride	Lower CI95% one-sided	Upper CI95% one-sided	Lower CI95% two-sided	Upper CI95% Two-sided
**Confidence Intervals for the “risk difference” HAP-Fluoride**						
ICDAS increase ≥ 2 in at least one tooth up to visit 4 (after 24 weeks)	ITT	− 4.1%	− 16.4%	7.9%	− 18.3%	10.1%
ICDAS increase ≥ 2 in at least one tooth up to visit 6 (after 48 weeks)	ITT	− 3.9%	− 16.1%	8.4%	− 18.5%	10.7%

**Figure 3:**
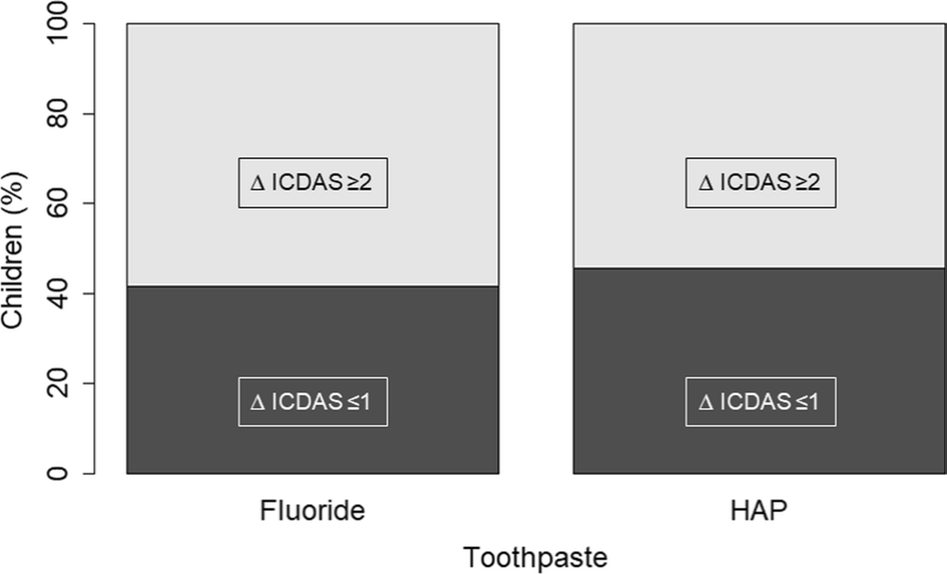
Increase in ICDAS ≥ ∆2 in the fluoride, and HAP-group. In both groups, the proportion of children who developed at least on one tooth a caries lesion of ICDAS ≥ ∆2 is not significantly different (more details are shown in Table 4).

The exact one-sided upper 95% confidence limits for the difference in proportion of subjects with ICDAS increase ≥ 2 up to visit (8.4%) were below the non-inferiority margin 20% [see primary endpoint] (PP analysis). This indicates that the test toothpaste (HAP) is non-inferior to the control toothpaste (fluoride) concerning this secondary endpoint (Fig. 3). This is also true for the exact two-sided upper 95% confidence limits. Superiority cannot be assumed as the confidence intervals are not below zero.

#### Plaque control record (PCR)

**Figure 4:**
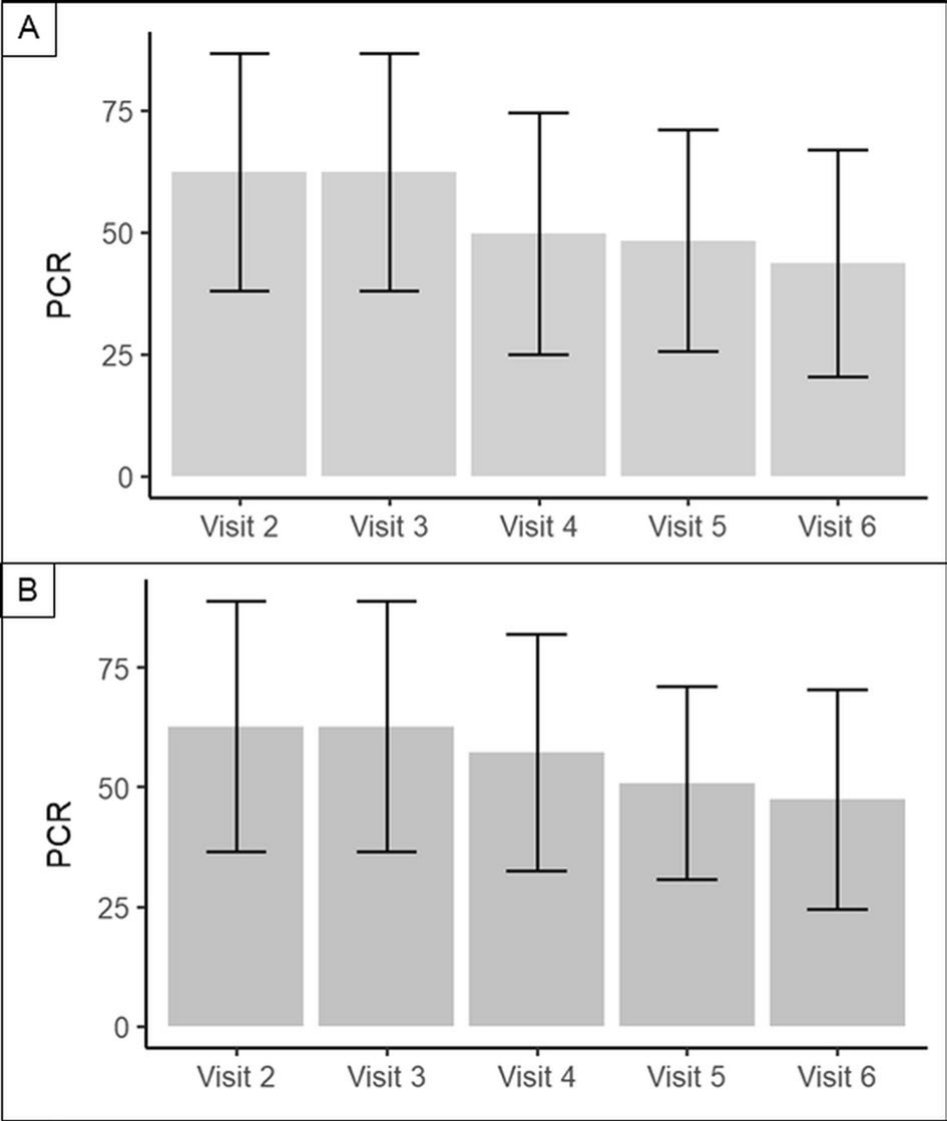
PCR-values from visit 2 to visit 6. A represents the HAP-group, while B shows the fluoride-group. In both groups the PCR-values decreased significantly (Friedman-test, p < 0.001). There was no difference between the HAP and the fluoride-group.

The distributions of the Plaque Control Record (PCR) differentiated by toothpaste at visit 2 (baseline) to visit 6 (end of study of study, after 336 days) are shown in Table 5. The results indicate that the PCR scores only slightly differed between treatment groups (HAP vs. fluoride) but decreased in both treatment groups from (V2) to the end of study (V6) (Fig. 4).

**Table 5:** Plaque Control Record (PCR) by toothpaste at visit 2 (baseline) to visit 6.

		Toothpaste	
		Hap	Fluoride
(V2) PCR index	Mean	62.4	62.7
	SD	24.3	26.2
	N	88	89
(V3) PCR index	Mean	62.4	62.7
	SD	24.3	26.2
	N	88	89
(V4) PCR index	Mean	49.8	57.2
	SD	24.8	24.7
	N	88	89
(V5) PCR index	Mean	48.3	50.8
	SD	22.8	20.2
	N	88	89
(V6) PCR index	Mean	43.7	47.5
	SD	23.3	23.0
	N	88	89

*SD* standard deviation

The results of ANCOVA indicate the change/reduction of PCR (V6-V2) was not significantly different for both toothpastes (p = 0.152).

An additional ANCOVA that includes the interaction term “treatment x center” showed that effects of both toothpastes were not different in the centers Poznan and Bialystok. Moreover, a non-parametric test (Friedman Test) was performed to analyze whether PCR (coverage of the assessed primary molars with bacterial plaque according to the criteria) decreased during the whole observation period (visit V2 to visit 6). The Friedman test revealed that in each treatment group (HAP and fluoride) the decrease in PCR during the observation period (visit 2 to visit 6) was significant (p < 0.001).

#### Modified gingival index (GI)

The modified Gingival Index (GI) was calculated as the mean of the modified GI scores of the eight included primary molars (teeth 54–85). Table 6 shows the descriptive statistics of modified GI score at visit V2 (baseline) to V6 (end of study) for both treatment groups/toothpastes. The results of ANCOVA indicate the change/reduction of modified GI (V6-V2) was not significantly different for both toothpastes (p = 0.853).

**Table 6:** Modified Gingival Index (GI) by toothpaste at visit 2 (baseline) to visit 6.

	Toothpaste	
	HAP	Fluoride
**(V2) Modified GI**		
Mean	.24	.29
SD	.30	.34
N	88	89
**(V3) Modified GI**		
Mean	.14	.19
SD	.22	.31
N	88	89
**(V4) Modified GI**		
Mean	.08	.12
SD	.18	.21
N	88	89
**(V5) Modified GI**		
Mean	.01	.02
SD	.06	.07
N	88	89
**(V6) Modified GI**		
Mean	.01	.02
SD	.07	.08
N	88	89

SD, Standard deviation.

**Figure 5:**
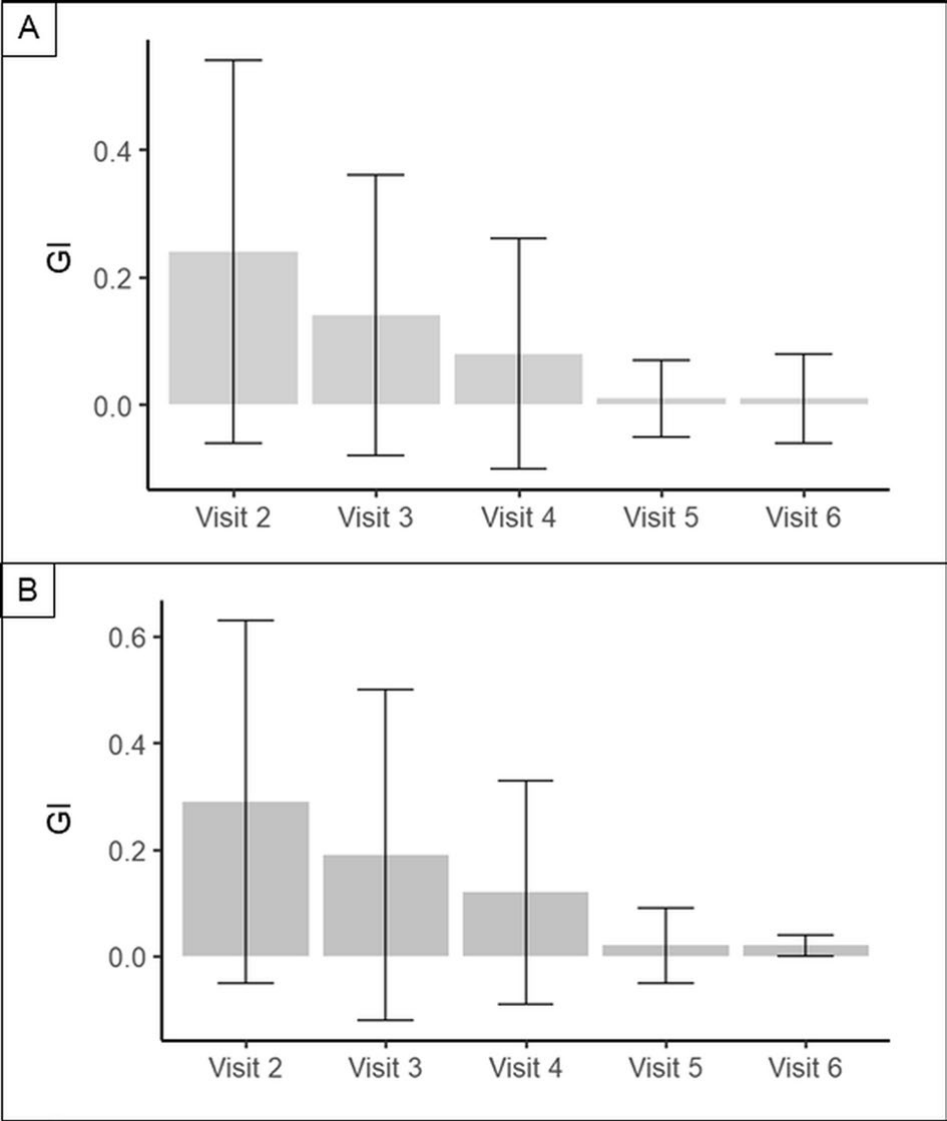
GI from visit 2 to visit 6. A represents the HAP-group, while B shows the fluoride-group. In both groups the GI decreased significantly. There was no difference between the HAP and the fluoride-group.

In almost all cases, the categories “absence of inflammation” (= 0) or “change in color of any portion but not the entire marginal or papillary unit” (= 1) were reported. In two cases, “change in color which involves the entire marginal or papillary unit” was reported and in no case “distinctly red”. The results on the GI are shown in Fig. 5.

### Inter-examiner reliability (ICDAS)

To test the inter-examiner reliability, linear and squared weighted Kappa coefficients were calculated (Table 7).

**Table 7:** Overview of linear and squared weighted Kappa coefficients as measures of interrater reliability (re-training 1 and 2 was performed during the course of the study).

No. of training	Mean of linear weighted Kappa coefficients	Mean of square weighted Kappa coefficients
1 (Initial training)	0.82	0.91
2 (Retraining 1)	0.84	0.93
3 (Retraining 2)	0.83	0.92

### Safety

No serious and no severe adverse events (AEs) were reported in course of the study. In total, 79 AEs were reported in 48 subjects:

- 48 AEs in 28 subjects who applied HAP (test toothpaste) and
- 31 AEs in 23 subjects who applied fluoride (control toothpaste).

Table 8 summarizes severity, causality, and outcome of AEs. There were no statistically significant differences between the toothpastes (p > 0.05, two-sided exact Fisher Test).

**Table 8:** Overview of severity, causality, and outcome of adverse events.

	Treatment					
	HAP		Fluoride		Total	
	n	%	n	%	n	%
**Severity**						
Mild	40	83.3%	28	90.3%	68	86.1%
Moderate	8	16.7%	3	9.7%	11	13.9%
Severe	0	0.0%	0	0.0%	0	0.0%
**Severe AE**						
No	48	100.0%	31	100.0%	79	100.0%
**Causality**						
Probable/likely	1	2.1%	3	9.7%	4	5.1%
Possible	5	10.4%	0	0.0%	5	6.3%
Unlikely	41	85.4%	28	90.3%	69	87.3%
Unrateable	1	2.1%	0	0.0%	1	1.3%
**Outcome**						
Ongoing	0	0.0%	2	6.5%	2	2.5%
Resolved	47	97.9%	28	90.3%	75	94.9%
Resolved with sequelae	1	2.1%	1	3.2%	2	2.5%
Death	0	0.0%	0	0.0%	0	0.0%
Lost to follow-up	0	0.0%	0	0.0%	0	0.0%

## Discussion

The persistent prevalence of ECC at a high level around the globe, despite the proven effectiveness of fluoride, justifies the need for other caries preventive materials that can be used by children of all ages at any concentration. The effectiveness of HAP to prevent caries development and promote caries remineralization has been demonstrated in several clinical studies^31,32,38,42^ This clinical trial was conducted to explore future proper clinical application of hydroxyapatite toothpastes. Thus, the present study investigated the non-inferiority of HAP toothpaste to fluoride toothpaste in children. As children swallow, intended or unintended, most of their toothpastes while brushing, this leads to an increased risk of developing (dental) fluorosis or other side effects. Therefore, an active ingredient is needed that is safe if swallowed and as effective as fluorides in caries prevention.

This is the second clinical trial on enamel caries development showing the non-inferiority of a fluoride-free HAP toothpaste compared to fluoride toothpastes with clinically proven caries-preventing effect^14,16,17^. Schlagenhauf et al*.* observed in a 6-month study in orthodontic patients an increase in enamel caries ICDAS code ≥ 1 in 56.8% (ITT) and 54.7% (PP) of the HAP group subjects compared with 60.9% (ITT) and 61.6% (PP) of the fluoride control group (with 1400 ppm fluoride)^31^. The non-inferiority of HAP compared to fluoride as confirmed in our study (both for the primary and secondary endpoints regarding ICDAS; Tables 2, 3 and 4) goes in line with the results of a recently published in situ study by Amaechi et al*.* that analyzed the remineralization efficacy of a fluoride-fee HAP toothpaste on initial caries lesions in human primary teeth^32^. Moreover, a placebo-controlled trial published in 1989 demonstrated a caries preventing effect of a hydroxyapatite containing toothpaste in Japanese school children^14,38^. The non-inferiority of the HAP toothpaste compared to a fluoride toothpaste with antibacterial counter-ions on PCR and GI as shown in Tables 5 and 6 was already reported in other clinical trials^35,36^.

Our results show that the development of new enamel caries lesion of ICDAS ≥ code 1 severity was observed in 73.4% of the subjects (Tables 2 and 3). This number seems very high but can be explained by the high prevalence of ECC in Poland and also by the fact that we used ICDAS to assess the caries status, i.e. non-cavitated caries lesions were included in the assessment. If perhaps we had used dmft index this number would be much lower. However, ICDAS was used in order to capture any developed lesion as early as possible. On the other hand, as expected, the proportion of subjects with development of at least one new enamel caries lesion with ICDAS ≥ code 2 up to visit 6 was lower (56.5%) than proportion of subjects with development of enamel caries lesions with ICDAS ≥ code 1 or the progression of an existing enamel caries lesion by at least ICDAS ≥ code 1. These findings are supported by data of Milsom et al*.*^59^. In that study, it was found that caries-active children have a 5–6 times higher incidence of new cavities compared to caries-free children. In our study we also chose caries-active children (the presence of a caries restoration (filling) on a minimum of 1 primary molar was one of the inclusion criteria) because of the very high prevalence of ECC in developed countries and especially developing countries^1,3,8–11^. Consequently, the caries risk of our study populations (i.e. a caries restoration on a minimum of 1 primary molar) represented the average caries risk within children. Thus, the HAP toothpaste was shown to be effective in a general population as well as in high caries-risk group such as orthodontic patients^31^. The PP and ITT analyses of the primary and secondary endpoint (ICDAS) of our study indicated slightly less, but not statistically different caries development in children that used HAP toothpaste compared to children that used fluoride toothpaste (Tables 2, 3 and 4). These results clearly confirmed the clinical non-inferiority of the HAP toothpaste compared to the fluoride toothpaste. The PP analysis showed that 27.3% children of the HAP group and 25.8% of the children in the fluoride group did not show any change in caries status on the evaluated primary molars during the course of the study.

It is well-known that the concentration of fluorides in toothpastes for children (especially for infants and toddlers) has always been balanced against the potential fluorosis-risk^16,21,22^. Hellwig et al*.* demonstrated in an in situ study that 500 ppm fluoride significantly remineralized initial caries lesions in deciduous enamel, and this remineralization effect could not be improved by higher fluoride concentrations (i.e. 1,000 and 1,500 ppm fluoride)^17^. These findings are supported by in vivo data by Biesbrock et al*.*^56^. Consequently, we chose as control a toothpaste with 500 ppm fluoride because this represents a good compromise between caries preventing efficacy and fluorosis risk for children of the control group. Furthermore, teeth were brushed three times a day in our study, thus three fluoride impulses (not only 2 × daily toothbrushing like recommended by most dentists) were ensured in our study. Finally, we chose amine fluoride (control toothpaste), a more efficient fluoride compound than e.g. sodium fluoride. Amine fluoride shows (in contrast to sodium fluoride or sodium monofluorophosphate) antibacterial properties due to the ammonium salt (i.e. the counterion of the fluoride)^52,54^. Taken together, this proves that the use of the control toothpastes with 500 ppm fluoride provided as amine fluoride has caries preventing effects on the primary dentition. Consequently, the results of our study demonstrated the caries preventive effects of a HAP toothpaste which is non-inferior to a fluoride toothpaste.

The amount of toothpaste used in our study was ‘pea-sized’ because of regulatory reasons on fluoride in toothpastes^62^. In contrast to fluoride toothpastes, the amount of HAP toothpastes does not have to be ‘pea-sized’, because of the high biocompatibility of HAP^48^. However, due to the double-blind study design, the HAP toothpaste could not be dosed higher than the fluoride toothpaste in our study. In principle, the caries preventing effect of the tested HAP toothpaste may be further increased by using higher amounts of this toothpaste. Fabritius-Vilpoux et al*.*, for example, demonstrated in an in vitro scanning electron study that the quantitative adhesion of HAP-particles to enamel surfaces can be increased by higher HAP concentrations in mouthwash formulations^34^.

The clinical non-inferiority of HAP compared to fluorides as shown both in the study of Schlagenhauf et al*.*^31^, and in our present study can be explained by different modes of action of HAP in the oral cavity. The modes of action of HAP are based on physical, bio-chemical, and biological principles (for details see e.g. Ref.^29^). HAP interacts with tooth surfaces and dental plaque^30,33,34,43,63,64^. Several in situ and in vitro studies have analyzed the modes of action of hydroxyapatite with respect to cavity protection. In situ and in vitro studies were able to show a remineralizing effect of HAP on dentin and enamel^32,39,45^. Here, TMR (transverse microradiography) was used to measure not only the mineral gain, but also a reduction in lesion depth when using a HAP-toothpaste^32,39,42,45^. Moreover, HAP significantly reduces bacterial colonization to the tooth surface *in situ*^30^. The effect of bacterial reduction to tooth-surfaces is comparable to chlorhexidine^30,40^. Furthermore, HAP is organized in microclusters, when formulated in toothpastes^34^. Those microclusters act as soft toothpaste abrasives^14,65^. Besides that, HAP has been shown to function as calcium- and phosphate-ion releasing active ingredient^33^. The same study shows its pH-buffering properties when present in acid-producing cariogenic biofilms^33,64,66^. Recent SEM (scanning electron microscopy) studies clearly confirm the attachment of the active ingredient HAP to the enamel surface as well as to dental materials^34,43^. Interestingly, Shaw et al*.*, for example, reported significantly higher calcium and phosphorus levels in dental plaque of caries-free children compared to caries-active children^67^. Consequently, the use of biomimetic HAP as a calcium-phosphate-reservoir in dental plaque of caries-active children seems to be a promising approach to improve tooth remineralization, to decrease the level of tooth demineralization, and thus to reduce the overall caries risk. The simplified chemical equations of HAP acting as a calcium-phosphate-reservoir under acidic conditions (cariogenic biofilms, erosive conditions due to dietary habits etc.) is^33^:

- Ca_5_(PO_4_)_3_(OH) + 7 H^+^  → 5 Ca^2+^  + 3 H_2_PO_4_^-^ + H_2_O.

Limiting factors of our study are as follows. In this study teeth were brushed with electric toothbrushes in the morning and in the evening by the parents in our study. Electric toothbrushes are known to be more efficient in plaque removal compared to manual toothbrushes^68^. On the other hand, the percentage of users of electric toothbrushes has increased within the last years, especially in developed countries. Since parents brushed the teeth of their child twice a day (the third brushing was performed by the children themselves under supervision of an adult), an influence of the age of the children (i.e. a possible improvement of motor skills within the study course) can be excluded.

A further limitation of our study is the unknown influence of the diet. It is well-known that sugar consumption and its frequency have a significant influence on caries development^13^. Here, we did not monitor the diet of each subject as the aim was to test two different toothpastes, but not the influence of the diet on the caries progress. Nevertheless, one may argue that we tested both, the HAP and the fluoride toothpaste, under real-life conditions due to the absence of any specific inclusion or exclusion criteria concerning the diet. However, we can assume that subjects had comparable dietary habits as both study centers are located in urban areas of Poland. A recently published meta-analysis on the nutrient intake by Polish pre-school children showed that the carbohydrate-intake is 24% higher than recommended^69^.

A strength of the study is the fact that the influence of other preventive measures (e.g. the use of antiseptics like e.g. chlorhexidine or professional tooth cleaning) were excluded in our study, i.e. the study focused exclusively on toothbrushing with toothpaste (HAP vs. fluoride).

Caries lesions were assessed with ICDAS, a state-of-the-art system for measuring caries, which was developed by an international team of caries researchers^50,51,70^. In contrast to the frequently used dmft index, ICDAS offers the advantage to evaluate also initial enamel caries lesions (i.e. caries at a non-cavitated stage)^50^; thus, compared to dmft index, more caries lesions can be assessed using ICDAS. Investigation of initial caries is of great importance, because these early caries lesions can be remineralized by dental care products for home use either with HAP or fluoride^17,32,42^. Moreover, in a combined in vivo-in vitro study the use of ICDAS was shown to be more suitable than DIAGNOdent Pen and CarieScan PRO in detecting and assessing occlusal caries in primary molars^70^. The calculation of interrater reliability in our study indicated a high interrater reliability of the ICDAS scoring throughout the course of our study. This was shown both by the linear weighted and in particular the squared weighted Kappa coefficients between the raters and the benchmark rater (an experienced cariologist) and among the raters themselves (Table 7). Nevertheless, further clinical studies might analyze the impact of HAP toothpastes on DMFS/DMFT [dmfs/dmft] indices, thus making the results also comparable to previously performed studies which focus on fluoride toothpastes^16^.

In general, due to the high risk for young children to develop caries in the primary dentition, as demonstrated in our study as well as previous studies, using a comprehensive caries-preventive approach, i.e. a combination of regular dental visits (at least every 6 months), thorough plaque removal by tooth brushing with electric toothbrush and toothpaste coupled with low sugar consumption^13^, and when necessary, the application of fissure sealants^71–73^, is well-suited for prevention or at least to significantly reduce the ECC-risk. These preventive measures are very important considering that it was been demonstrated that caries in the primary dentition of 7-year-old Polish children increased the risk for the development of caries in the permanent dentition for more than 5 times^8^.

## Conclusions

In children, the impact of the daily use of a toothpaste with microcrystalline HAP on enamel caries progression in the primary dentition is not inferior to that of a fluoride control toothpaste. Thus, the active ingredient hydroxyapatite is a biomimetic alternative to fluorides in toothpastes for children. Unlike fluorides (e.g. risk of dental fluorosis), HAP has a high biocompatibility and is safe if accidentally swallowed.
